# Wealth-based equity in essential newborn care practices in Ethiopia: A cross-sectional study

**DOI:** 10.1371/journal.pone.0338193

**Published:** 2025-12-05

**Authors:** Tadesse Guadu Delele, Lars Åke Persson, Kassahun Alemu Gelaye, Joanna Schellenberg, Seblewengel Lemma, Atkure Defar, Theodros Getachew, Girum Taye, Solomon Shiferaw, Zewditu Abdissa, Amare Tariku, Meseret Zelalem, Della Berhanu

**Affiliations:** 1 Institute of Public Health, College of Medicine and Health Sciences, University of Gondar, Gondar, Ethiopia; 2 Department of Disease Control, Faculty of Infectious and Tropical Diseases, London School of Hygiene & Tropical Medicine, London, United Kingdom; 3 Health System and Reproductive Health Research Directorate, Ethiopian Public Health Institute, Addis Ababa, Ethiopia; 4 Department of Epidemiology & Biostatistics, Institute of Public Health, College of Medicine and Health Sciences, University of Gondar, Gondar, Ethiopia; 5 School of Public Health, Addis Ababa University, Addis Ababa, Ethiopia; 6 Department of Maternal, Child Health, Adolescent and Nutrition, Ministry of Health, Addis Ababa, Ethiopia; 7 Department of Pediatrics and Child Health, School of Medicine, College of Medicine and Health Sciences, University of Gondar, Gondar, Ethiopia; Donor Organization in Nepal, NEPAL

## Abstract

**Background:**

The World Health Organization has listed several newborn care practices as essential for health and survival. Reports from low-income countries, including Ethiopia, have shown inequities in these practices, but their link to place of birth remains insufficiently explored. We aimed to analyze the wealth-based equity of selected essential newborn care practices, i.e., skin-to-skin care, delayed bathing, proper cord care, and timely breastfeeding initiation, among neonates born in health facilities and homes in Ethiopia.

**Methods:**

The Performance Monitoring for Action Ethiopia 2019–2020 survey was conducted in five regions, representing 90% of the country’s population, and included data on 2,493 newborns. Household wealth quintiles were derived using principal component analysis of asset ownership. We analyzed the wealth-based equity of selected essential newborn care practices for facility and home deliveries using equiplot, equity gaps, equity ratios, and concentration indices.

**Results:**

Overall, skin-to-skin care and delayed bathing showed minimal inequities among babies born in health facilities. When comparing the extreme wealth groups, minor socio-economic differences were observed in delayed bathing and timely breastfeeding initiation. When wealth was treated as a continuous variable across all respondents, delayed bathing and proper cord care were more common in better-off households. For home births, the equiplots showed that all selected essential newborn care practices were more frequent among the least poor groups. While comparing the extreme wealth groups, socio-economic inequities were evident in skin-to-skin care and delayed bathing practices. When wealth was considered a continuous variable, skin-to-skin care and delayed bathing were more common among better-off households.

**Conclusion:**

The coverage of selected essential newborn care practices was higher in facility deliveries. There were minimal socio-economic differences in newborn care for facility births, while inequities in skin-to-skin care and delayed bathing were prominent in home births. Initiatives should aim to enhance equity in newborn care within health facilities while promoting facility deliveries and improving newborn care practices at home to achieve equitable newborn care in Ethiopia.

## Background

The 2030 Sustainable Development Goal (SDG) 3.2 aims to reduce neonatal mortality to 12/1000 live births or less [1]. Despite global progress, evidence from low- and middle-income countries reveals persistent within-country inequities in essential newborn care for facility births, but especially home births [2]. Addressing these inequities is critical for achieving the SDG targets in sub-Saharan Africa, where neonatal mortality remains disproportionately high [3].

Promoting essential newborn care practices both at health facilities and in home settings is a proven strategy to improve newborn survival [4]. The World Health Organization (WHO) recommends several key practices—thermal care through skin-to-skin contact and delayed bathing for at least 24 hours, clean cord care with chlorhexidine application, and initiation of breastfeeding within the first hour after birth [5]. These interventions are simple, evidence-based, and cost-effective; however, their adoption remains uneven across socioeconomic groups and delivery settings.

In Ethiopia, several national initiatives have been implemented to enhance equitable access to quality newborn care. These include the Health Extension Programme, the Women’s Development Groups, the Community-Based Newborn Care (CBNC) initiative, and the community-based health insurance scheme. These programs have contributed to increased facility deliveries and improved newborn care practices [6–8]. Nonetheless, the extent to which these gains are equitably distributed across wealth groups is not fully understood.

Recent nationally representative survey data (2019–2020) show that neonates born in health facilities receive markedly higher coverage of essential care practices compared with home births—skin-to-skin care (76% vs. 9%), delayed bathing until 24 hours after birth (73% vs. 39%), proper cord care (89% vs. 65%), and first-hour initiation of breastfeeding (69% vs. 62%) [9]. While earlier studies based on the 2016 and 2019 Ethiopian Demographic and Health Survey (DHS) and community-based surveys have indicated some wealth-related disparities—particularly in skin-to-skin care and delayed bathing practices—they were limited by long recall periods, which can introduce recall bias and reduced accuracy [10–12].

What remains unclear, however, is whether wealth-based inequities in essential newborn care practices persist when assessed using data with a short recall period. This gap is critical because understanding the magnitude and pattern of inequities under improved measurement conditions can better inform targeted policy responses [13]. The Performance Monitoring for Action (PMA) 2019–2020 survey, with its shorter seven-week recall period, provides a unique opportunity to generate more accurate and timely estimates [9]. Therefore, this study aimed to investigate wealth-based inequities in selected essential newborn care practices among Ethiopian infants born at health facilities and homes, using both absolute and relative measures of equity [14].

## Methods

### Ethics approval and consent to participate

PMA Ethiopia received ethical approval from Addis Ababa University, College of Health Sciences (AAU/CHS) (Ref: AAUMF 01–008), and the Johns Hopkins University Bloomberg School of Public Health (JHSPH) Institutional Review Board (FWA00000287). Written informed consent was obtained from all study participants. For mothers under 18 years of age, assent was also obtained from their partner or another family member.

### Setting

The study was conducted in five Ethiopian regions—Tigray, Afar, Amhara, Oromia, Southern Nations, Nationalities, and Peoples’ (SNNP)—as well as the Addis Ababa city administration [15]. Together, these areas represent 90% of the country’s total population. Ethiopia’s estimated population is around 120 million, with approximately 80% residing in rural areas [16]. The country has a three-tiered health system: 1) primary care consisting of health posts, health centers, and primary hospitals; 2) secondary care with general hospitals and specialty clinics; and 3) tertiary care with specialized hospitals [17].

### Data source, sampling, and quality control

This study used secondary data from the Ethiopian Performance Monitoring for Action (PMA) survey conducted in 2019–2020. The survey collected data using a standardized questionnaire on key maternal and newborn health indicators, administered via the Open Data Kit software (University of Washington, USA).

Data were collected by trained interviewers or resident enumerators who had a bachelor’s degree in health sciences. Field supervisors and regional coordinators oversaw data collection to ensure quality. Detailed descriptions of the PMA survey methodology have been published elsewhere [15].

A total of 2,493 newborns were included. The survey applied a two-stage cluster sampling design stratified by urban and rural residence and by region. In total, 217 enumeration areas (EAs) were selected using probability proportional to population size, and 35 households were randomly selected within each EA. Pregnant and postpartum women were invited to participate.

Interviews were conducted approximately seven weeks after birth (median: 7.4 weeks, interquartile range 6.9 weeks) to minimize recall bias. This time allowed mothers to complete the immediate postpartum period while still accurately recalling newborn care practices their infants received. Consenting women were interviewed using a structured questionnaire that covered sociodemographic characteristics (age, education, region, religion, residence, and household wealth), parity, place of delivery, and newborn care practices performed either by healthcare staff (in facilities) or by family members or assistants (at home).

### Outcome variables and measurements

The selected outcome variables included thermal care (skin-to-skin care and delayed bathing), cord care practice, and breastfeeding initiation in the first hour. These practices were assessed based on the WHO guidelines for essential newborn care, as reported by the mother [5].

Skin-to-skin care was coded as “Yes” if the mother reported that the neonate was placed naked on her chest against her skin immediately after delivery and “No” otherwise.

Delayed bathing was coded as “Yes” if the mother reported washing her newborn was postponed until at least 24 hours after delivery and “No” if otherwise.

Proper cord care was coded “Yes” if the mother reported not applying anything or only chlorhexidine on the cord stump after cutting and “No” if otherwise.

Timely initiation of breastfeeding was coded as “Yes” if the mother reported that she put the baby to her breast within an hour after birth and “No” if otherwise.

The selected essential newborn care practices were stratified by delivery place and household wealth. The household wealth was assessed based on asset ownership, including electricity, television, radio, watches, telephones, and refrigerators. The wealth index for each household was constructed based on a principal component analysis—a statistical method that combines information on household assets into a single measure of wealth—expressed as wealth quintiles ranging from 1 (poorest) to 5 (richest).

We used these quintiles to construct the equiplots, equity gaps, and equity ratios, while the continuous wealth index was applied in the concentration index—a measure showing whether newborn care practices are more common among the poor or the rich—and the slope index. We chose these equity measures because they capture both absolute gaps and relative differences in essential newborn care practices across wealth groups, providing a more comprehensive picture of inequities. The absolute measures capture the actual size of the gap in essential newborn care practices between the poorest and richest groups, while the relative measures show the proportional difference.

### Analyses

Participation was displayed in a study flow diagram. Characteristics of households, mothers, and neonates were cross-tabulated against the place of delivery. The equity of the selected essential newborn care practices was calculated using four standard equity measures, separately for births at home and in health facilities: equiplot, equity gaps, equity ratios, and concentration indices [14].

Sampling weights were applied to assure the regional representativeness of the data, and clustering was accounted for in all analyses [18]. The equity of selected essential newborn care practices was illustrated using equiplots to show gaps across household wealth quintiles among neonates born at health facilities and homes [14]. The equity gap is the absolute percentage point difference in service coverage between the extreme groups. In this case, the gap was calculated between the highest and lowest wealth quintiles. Similarly, the wealth equity ratio was calculated by dividing the essential newborn practice coverages between mothers in the highest household wealth quintile by the coverages among mothers in the lowest household wealth quintile.

The concentration index measures the cumulative percentage of the population ranked by household wealth against the cumulative percentage of each newborn care practice [14,19]. A value of zero indicates no socioeconomic-related inequity, while negative and positive values show higher coverage among the poorest and richest groups, respectively. Similarly, the slope index of inequity (SII) estimates inequities across the full wealth spectrum [19], with zero indicating no inequity, negative values favoring the poorest, and positive values favoring the richest. The concentration index (CIX) and slope index of equity (SII), including standard errors and p-values, were assessed using commands downloaded from the International Center for Equity in Health [14,19]. Data analyses were performed using STATA version 18 software. Weighted proportions of both the outcome and explanatory variables were used in all analyses.

## Results

### Study participation

**Fig 1:**
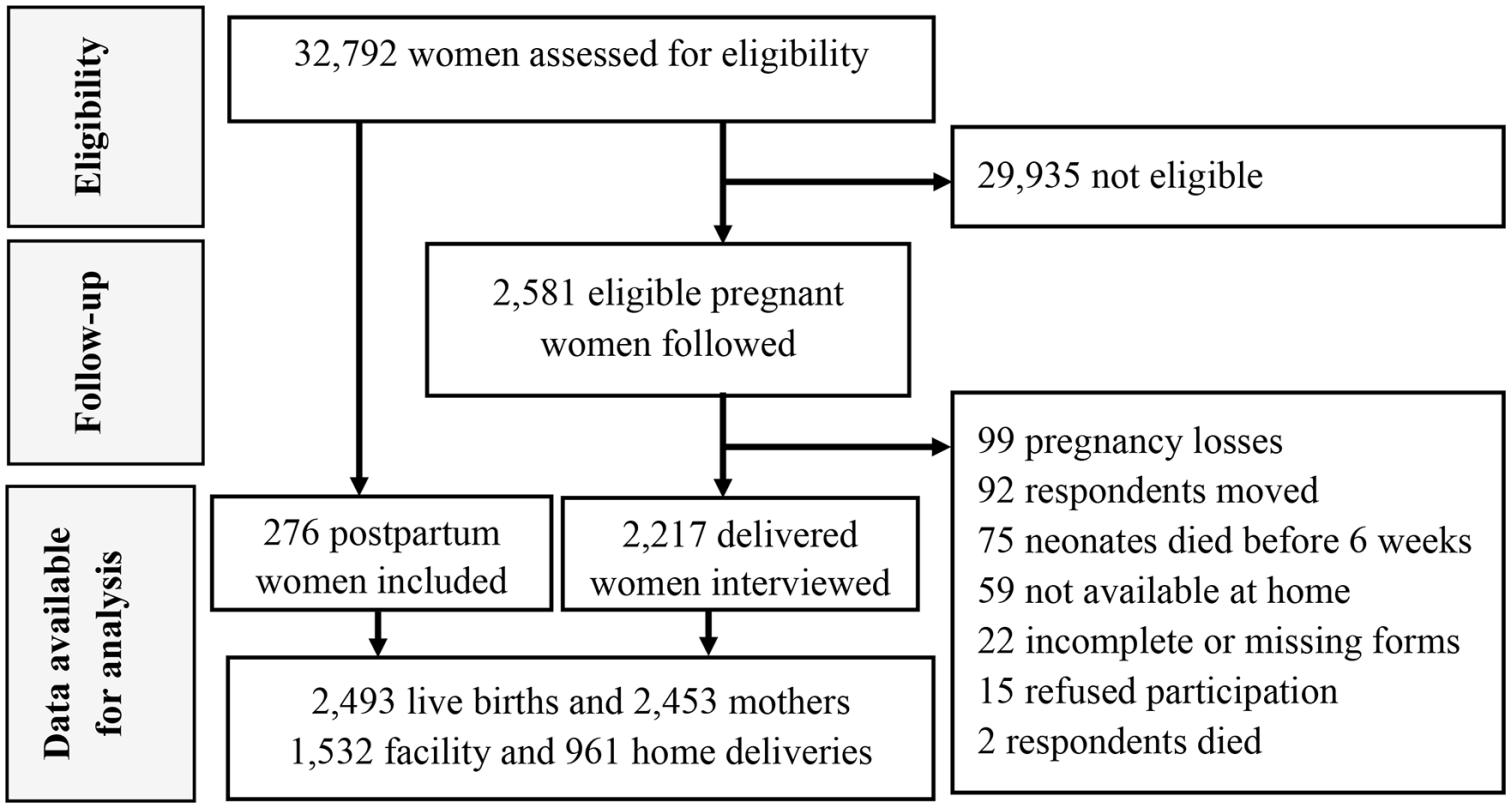
Study flow. PMA Ethiopia 2019-2020.

A total of 32,792 women were assessed for eligibility. Those who were pregnant or within six weeks postpartum, permanent residents of the households, and consented to participate were included in the study. Of these, 2,581 women were eligible, and after omissions, 2,453 mothers were included (95%). They had 2,493 live births, including 40 twins (Fig 1). More than half (53%) of the neonates were born at health facilities.

### Sociodemographic characteristics

Facility delivery was markedly higher among urban women (92%) than rural women (43%) (Table 1). Nine out of ten women from the highest wealth quintile and three out of ten in the lowest quintile delivered in health facilities. A higher proportion of young women and those having their first baby delivered in health facilities. Most (85%) of the mothers who had a secondary education or higher level gave birth at health facilities, whereas 38% of those with no formal education did so. The place of delivery was similar among boys and girls.

**Table 1:** Sociodemographic characteristics of mothers. Ethiopian Performance Monitoring for Action survey 2019-2020, weighted data.

Characteristics	Category	n	Place of delivery, % (95% CI)	
			Health facility (N = 1532)	Home (N = 961)
Region	Tigray	421	70 (56, 82)	30 (18, 44)
	Afar	202	17 (6, 40)	83 (60, 94)
	Amhara	433	57 (47, 67)	43 (33, 53)
	Oromia	618	49 (39, 60)	51 (41, 61)
	SNNP	574	48 (37, 60)	52 (40, 63)
	Addis Ababa	245	98 (95, 99)	2 (1, 6)
Residence	Urban	966	92 (87, 96)	8 (3, 17)
	Rural	1,527	43 (36, 49)	57 (51, 64)
Wealth quintiles	1 Poorest	443	28 (22, 36)	72 (64, 78)
	2	377	39 (32, 46)	61 (54, 68)
	3	380	50 (42, 58)	50 (42, 58)
	4	468	58 (48, 68)	42 (32, 52)
	5 Least poor	822	94 (88, 97)	6 (2, 15)
Age (years)	15-19	218	57 (48, 67)	43 (33, 52)
	20-34	1,892	55 (49, 61)	45 (39, 51)
	35-49	380	44 (36, 52)	56 (48, 64)
Education	None	945	38 (32, 44)	62 (56, 68)
	Primary	899	56 (48, 62)	44 (38, 52)
	Secondary or higher	646	85 (79, 90)	15 (10, 21)
Previous live births	None	470	71 (70, 83)	23 (17, 30)
	1–3	1,329	56 (50, 63)	44 (37, 50)
	≥ 4	691	36 (30, 43)	64 (57, 70)
Sex of the baby	Boy	1,262	54 (47, 60)	46 (40, 53)
	Girl	1,231	53 (47, 59)	47 (41, 53)

SNNP = Southern Nations, Nationalities, and Peoples.

### Wealth-based equity in essential newborn care for facility births

Among facility-born neonates, 76% were put naked skin-to-skin on their mothers’ breasts, 73% had delayed bathing until at least 24 hours after birth, 89% had proper cord care, and 69% initiated breastfeeding initiation during the first hour.

**Fig 2:**
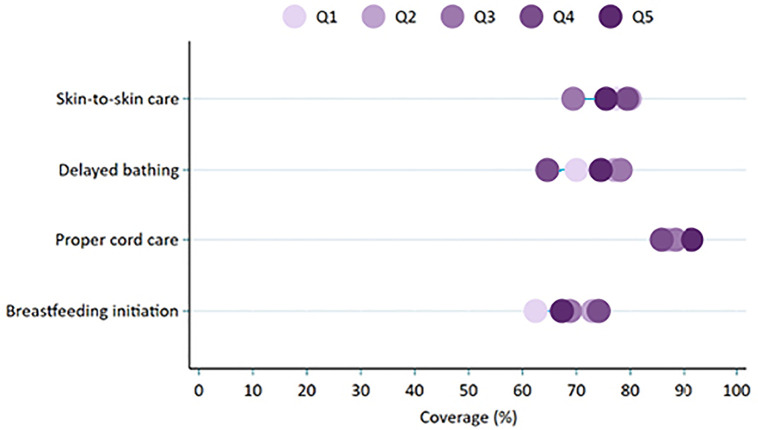
Wealth-based equity in skin-to-skin care, delayed bathing, proper cord care, and breastfeeding initiation among facility delivered neonates, PMA Ethiopia 2019-2020 survey.

Overall, for babies born in health facilities, the equiplots indicated little to no variations across wealth quintiles in skin-to-skin care, delayed bathing until at least 24 hours after birth, proper cord care, and timely breastfeeding initiation (Fig 2). Nonetheless, comparisons between the poorest and wealthiest groups revealed minor socio-economic differences in these practices (Table 2). Furthermore, when household wealth was considered as a continuous variable, delayed bathing and proper cord care were modestly more common among infants from better-off households.

**Table 2:** Wealth-based equity in skin-to-skin care, delayed bathing, proper cord care, and breastfeeding initiation practices in facility and home-delivered neonates. Ethiopian Performance Monitoring for Action survey 2019-2020.

Newborn care practices	Q1%	Q5%	Difference (Q5-Q1; % points)	Ratio (Q5:Q1) value	Slope index of inequity/SII			Concentration index/CIX		
					Coefficient	SE	P-value	Coefficient	SE	P-value
**Facility deliveries**										
Skin-to-skin care	77	76	−1	1	−0.1	0.04	0.2	−0.01	0.01	0.2
Delayed bathing	70	75	5	1	0.11	0.04	0.001	0.03	0.01	0.001
Proper cord care	92	92	0	1	0.1	0.03	0.002	0.02	0.01	0.002
Breastfeeding initiation	62	67	5	1	−0.04	0.04	0.4	−0.01	0.01	0.4
**Home deliveries**										
Skin-to-skin care	9	30	21	3	0.1	0.04	0.02	0.2	0.1	0.01
Delayed bathing	38	63	25	2	0.12	0.1	0.02	0.1	0.02	0.03
Proper cord care	67	72	5	1	−0.1	0.1	0.11	−0.02	0.01	0.1
Breastfeeding initiation	62	77	15	1	0.01	0.1	0.9	0.001	0.02	0.9

Q1 = Quintile 1, Q5 = Quintile 5, SE = Standard error

### Wealth-based equity in essential newborn care for home births

Among home-born neonates, 9% were placed skin-to-skin, 39% had their bathing delayed for at least 24 hours, 65% got proper cord care, and 62% got timely breastfeeding initiation.

**Fig 3:**
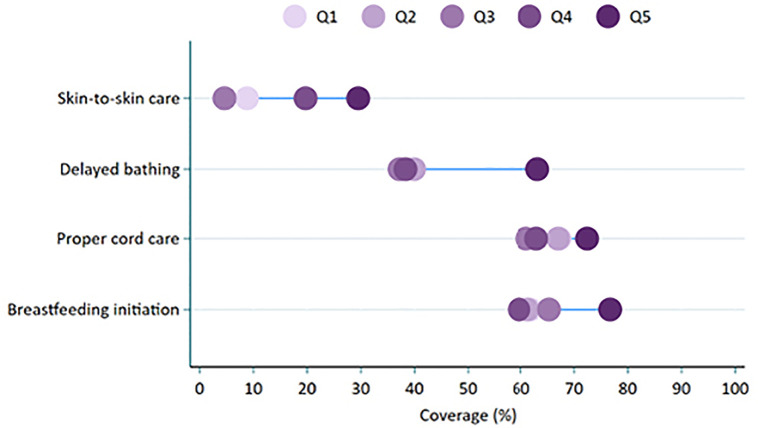
Wealth-based equity in skin-to-skin care, delayed bathing, proper cord care, and breastfeeding initiation among home-delivered neonates, PMA Ethiopia 2019-2020 survey.

The equiplots showed that skin-to-skin care, delayed bathing, proper cord care, and timely breastfeeding initiation differed across wealth groups, with these practices being more common in the least poor groups (Fig 3). The socioeconomic differences in proper cord care were minimal. When comparing the extreme groups, visible socio-economic inequities were reported in the practices of skin-to-skin care and delayed bathing (Table 2). Using household wealth as a continuous variable, skin-to-skin care and delayed bathing were more common in better-off households.

## Discussion

Overall, the coverage of selected essential newborn care practices was higher in facility deliveries than home births. This finding aligns with evidence from Ethiopia and other low- and middle-income countries, where facility births are associated with improved adherence to recommended newborn care practices [4,20,21]. Almost all women in the highest household wealth quintile and only three out of ten in the lowest quintile gave birth in a health facility, revealing persistent socio-economic inequities in access to skilled delivery care. Similar patterns have been observed across Sub-Saharan Africa and South Asia [22–24].

Among facility-born neonates, the coverage of selected essential newborn care practices—such as early skin-to-skin care, delayed bathing, proper cord care, and timely initiation of breastfeeding—was generally higher, though not optimal. This is consistent with studies in Ethiopia and elsewhere that reported incomplete implementation of WHO-recommended postnatal care standards within health facilities [25–27]. Our findings confirm the absence of wealth-based inequities in facility deliveries, as healthcare providers usually provide standardized care regardless of socio-economic status. Nonetheless, modest disparities were observed in delayed bathing and proper cord care—practices that often occur after discharge and thus depend on family behaviors and home environments. In Ethiopia, most mothers are discharged from the facility within 24 hours after birth [25], which increases the likelihood of early bathing contrary to WHO recommendations to delay the first bath by at least 24 hours to prevent hypothermia [28]. Strengthening discharge counseling on newborn thermal care, safe cord management, and postnatal follow-up is therefore essential to sustain facility-based gains in newborn health.

In contrast, the coverage of the selected essential newborn care practices was lower and more inequitable among home births. Wealth-based inequities were evident in skin-to-skin care and delayed bathing, favoring infants from better-off households. This finding is consistent with earlier Ethiopian and South Asian studies that highlight gaps in essential newborn care awareness and practice among the poorest families [4,29]. The high proportion of home deliveries and low coverage of thermal care among the poorest households underscore the persistent challenge of ensuring equitable and universal newborn care [5,30]. Strengthening the health extension program to promote facility deliveries, alongside improving antenatal counseling and birth preparedness for mothers likely to deliver at home, remains a critical strategy. Evidence from Malawi and Uganda shows trained community health workers and home-based counseling effectively improve newborn care behaviors and reduce inequities [31,32].

Our analysis also revealed that proper cord care practices, although suboptimal for home deliveries, were relatively equitable across wealth groups. This could indicate that cord care messages have been widely disseminated through both facility and community channels. However, timely breastfeeding initiation among home-delivered neonates showed notable wealth-based disparities, favoring infants from the wealthiest households. This pattern has been observed elsewhere in Sub-Saharan Africa, where differences in maternal literacy, exposure to counseling, and support from skilled attendants contribute to inequitable breastfeeding practices [4,33,34]. To address these gaps, community-based interventions—such as early home visits by health extension workers, peer counseling, and breastfeeding education—should be scaled up to reach disadvantaged mothers.

Lastly, our findings confirm that improving antenatal care (ANC) is central to advancing equity in newborn health outcomes. A previous article from the same study population reported that while 70% of pregnant women had at least one antenatal care (ANC) visit, only 40% completed four or more visits [35]. Increasing both the frequency and the quality of ANC is crucial, as evidence from multi-country analyses shows that ANC counseling on birth preparedness, facility delivery, and essential newborn care significantly improves neonatal outcomes [36,37]. Strengthening ANC content to ensure comprehensive education, combined with active follow-up by community health workers, would therefore enhance the continuum of care and reduce inequities, regardless of the place of birth.

### Strengths and limitations

The study participants were drawn from five regions and one city administration, covering about 90% of Ethiopia, which supports the generalizability of the findings to the national level. We only included a selection of the care components that the WHO classifies as essential; however, these selected indicators are among the most critical for newborn health and survival. Household wealth was used as the main socio-economic stratifier to examine equity in essential newborn care practices for infants born both at health facilities and at home. Other factors—such as maternal education, household location, income, and distance to health services—may also influence newborn care behaviors and contribute to disparities that were not captured in this analysis.

This study’s cross-sectional design limits causal inference, as associations observed between wealth status and newborn care practices do not imply causation. Furthermore, self-reported data from mothers may be subject to recall and reporting bias, particularly for facility-based births where some care practices might not have been directly observed by the mother. To mitigate this, data were collected when the infants were about seven weeks old, reducing the recall period and thus minimizing memory-related errors. Finally, the quality and consistency of care practices—such as whether recommended procedures were performed correctly or by trained personnel—were not assessed due to data limitations. Future research could complement these findings using longitudinal or observational designs that incorporate quality of care assessments and multiple dimensions of equity.

## Conclusion

The coverage of selected essential newborn care practices was higher in facility deliveries. For home births, skin-to-skin care and delayed bathing practices were particularly low. Minimal socioeconomic differences were observed in facility deliveries, whereas inequities in skin-to-skin care and delayed bathing were evident among home deliveries. It is essential to enhance facility deliveries and improve counseling of mothers at discharge, enabling them to continue providing proper care for their newborns at home.

## Supporting information

S1 Table Sociodemographic characteristics of mothers, PMA Ethiopia 2019–2020 survey, unweighted data. (DOCX)
