# Supplementary material for: Wealth-based equity in essential newborn care practices in Ethiopia: A cross-sectional study
Source: PLoS One. 2025 Dec 5;20(12):e0338193. doi: 10.1371/journal.pone.0338193 (PMC12680262; doi:10.1371/journal.pone.0338193)
Supplement: S1 Table — (DOCX) [file pone.0338193.s001.docx]

**Competing interests**

The authors declare that they have no competing interests.

**Funding**

This research was funded by the Bill and Melinda Gates Foundation (INV-009691 and INV-010320). The funder had no role in the design, data collection, analysis or interpretation of the data.

**Supplementary Table 1**. Sociodemographic characteristics of mothers, PMA Ethiopia 2019-2020 survey, unweighted data.

| Characteristics | Category | n | Place of delivery, n (%) (95% CI) | |
| --- | --- | --- | --- | --- |
|  |  |  | **Health facility**  **(N=1532)** | **Home**  **(N=961)** |
| Region | Tigray | 421 | 342 (81) (77, 85) | 79 (19) (15, 23) |
|  | Afar | 202 | 37 (18) (14, 24) | 165 (82) (76, 86) |
|  | Amhara | 433 | 262 (61) (56, 65) | 171 (39) (35, 44) |
|  | Oromia | 618 | 350 (57) (53, 61) | 268 (43) (39, 47) |
|  | SNNP | 574 | 302 (53) (49, 57) | 272 (47) (43, 51) |
|  | Addis Ababa | 245 | 239 (98) (95, 99) | 6 (2) (1, 5) |
| Residence | Urban | 966 | 904 (94) (92, 95) | 62 (6) (5, 8) |
|  | Rural | 1,527 | 628 (41) (39, 44) | 899 (59) (56, 61) |
| Wealth quintiles | 1 Lowest | 443 | 118 (27) (23, 31) | 325 (73) (69, 77) |
|  | 2 Lower | 377 | 147 (39) (34, 44) | 230 (61) (56, 66) |
|  | 3 Middle | 380 | 184 (48) (43, 54) | 196 (52) (47, 57) |
|  | 4 Higher | 468 | 292 (62) (58, 67) | 176 (38) (33, 42) |
|  | 5 Highest | 822 | 791 (96) (95, 97) | 31 (4) (3, 5) |
| Age (years) | 15-19 | 218 | 131 (60) (53, 66) | 87 (40) (34, 47) |
|  | 20-34 | 1,892 | 1196 (63) (61, 65) | 696 (37) (35, 39) |
|  | 35-49 | 380 | 205 (54) (49, 59) | 175 (46) (41, 51) |
| Education | None | 945 | 354 (37) (34, 41) | 591 (63) (59, 66) |
|  | Primary | 899 | 584 (65) (62, 68) | 315 (35) (32, 38) |
|  | Secondary or higher | 646 | 594 (92) (90, 94) | 52 (8) (6, 10) |
| Previous live births | None | 470 | 384 (82) (78, 85) | 86 (18) (15, 22) |
|  | 1 – 3 | 1,329 | 874 (66) (63, 68) | 455 (34) (32, 37) |
|  | ≥ 4 | 691 | 274 (40) (36, 43) | 417 (60) (57, 64) |
| Sex of the neonate | Boy | 1,262 | 790 (63) (60, 65) | 472 (37) (35, 40) |
|  | Girl | 1,231 | 742 (60) (58, 63) | 489 (40) (37, 42) |

SNNP=Southern Nations, Nationalities and Peoples’
